# Frailty is associated with an increased risk of postoperative pneumonia in elderly patients following surgical treatment for lower-extremity fractures: A cross-sectional study

**DOI:** 10.1097/MD.0000000000033557

**Published:** 2023-04-14

**Authors:** Yili Ou, Hong Wang, Ling Yang, Wei Jiang

**Affiliations:** a Department of Orthopedics, Deyang People’s Hospital/Orthopedic Center of Deyang City, Deyang, China.

**Keywords:** clinical frailty scale, elderly patient, frailty, lower-extremity fractures, postoperative pneumonia, risk

## Abstract

Elderly patients with lower-extremity fractures are at high risk of postoperative pneumonia (POP) after surgery due to longtime bed rest. This study aimed to explore whether preoperative frailty is an independent risk factor for POP in elderly patients undergoing surgical treatment for lower-extremity fractures. The study adopted a cross sectional design with 568 patients (≥60 years) admitted to a tertiary hospital in China from January 1, 2021 to June 30, 2022, for surgical intervention of a significant lower-extremity fracture. Preoperative frailty was assessed using the CFS (Clinical Frailty Scale). POP was assessed based on the classic diagnostic criteria reported in previous studies. Univariate and multiple logistic regression analyses were conducted to determine the impacts of preoperative frailty on POP. Of the 568 elderly patients, 65 (11.4%) developed pneumonia during postoperative hospitalization. There were significant differences among gender, hypoproteinemia, type of anesthesia, history of chronic obstructive pulmonary disease (COPD), and CFS scores. Multiple regression analysis revealed that the risk of POP in vulnerable, mildly frail, and severely frail patients increased by 2.38 times (*P* = .01, 95% CI [1.22–1.91]), 3.32 (*P* = .00, 95% CI [2.39–5.61]), and 5.36 (*P* = .00, 95% CI [3.95–6.52]), significantly. 12.8% of patients with hip fractures and 8.9% of patients with other main types of lower-extremity fractures developed POP. However, the difference between hip and non-hip fractures was not statistically significant (*P* > .05). Preoperative frailty increases the risk of POP in elderly patients after surgical treatment of main lower-extremity fractures. The severer the preoperative frailty is, the higher the risk of preoperative pneumonia is in elderly patients with lower-extremity fractures. CFS is simple and feasible for the assessment of frailty in elderly patients with lower-extremity fractures. Preoperative frailty assessment and appropriate management strategies should be considered in the perioperative management of elderly patients with lower-extremity fractures.

## 1. Introduction

Pneumonia is a research focus of postoperative complications in elderly patients.^[1,2]^ Many clinical studies have been conducted to prevent postoperative pneumonia (POP); however, the effect was not satisfactory. There are also many studies exploring the risk factors of POP in elderly patients, such as partial pressure of oxygen level at admission,^[3]^ age, sex, malnutrition and comorbidity.^[4,5]^ However, most of these variables are not modifiable, and we cannot intervene in these factors in a short time. Surgeons hope to find a variable that can more effectively predict the risk of POP in elderly patients so as to screen high-risk patients more scientifically and effectively.

A literature review found that almost all of the studies analyzing POP in elderly patients with lower-extremity fractures did not consider patient frailty which has been proved correlated with postoperative complications and even with greater influence on POP than that of age, sex, type of anesthesia, comorbidities, preoperative waiting time, etc.^[4,6,7]^ In recent years, with the in-depth study of frailty, there have been an increasing number of reports on the correlation between preoperative frailty and postoperative complications in elderly patients; however, few reports have focused on the association between preoperative frailty and POP. In rare relevant studies, preoperative frailty has been reported to be significantly correlated with pneumonia after major abdominal surgery.^[8]^ There are also reports in the field of orthopedics trauma. Shen Y used modified Frailty Index to review the correlation between patients’ frailty and POP and pointed out that the incidence of POP in elderly patients with hip fracture was 12.3%, and the overall risk of POP in frail patients with hip fracture was 2.08 times than that in patients without frailty.^[9]^ However, in elderly patients with lower-extremity fractures, the relationship between preoperative frailty and POP is uncertain, especially in patients with non-hip fractures. As is well known, except for hip fracture, elderly patients with lower-extremity fractures cannot bear weight with the injured limb after surgery and often need to stay in bed for a long time, so they are at a high risk of POP. The Clinical Frailty Scale (CFS) has been confirmed to correlate with POP in patients with hip fracture, and it is highly operational and effective in clinical application.^[10]^ However, it has not been used in elderly patients with non-hip lower-extremity fractures. Therefore, our primary purpose was to conduct a cross-sectional study to investigate the relationship between preoperative frailty (assessed by CFS) and POP in elderly patients with lower-extremity fractures and observe whether frailty plays a different role in POP in elderly patients with lower-extremity fracture (hip vs non-hip fracture). The second aim was to exam the effectiveness of CFS in screening frail patients with lower-extremity fracture.

## 2. Methods

### 2.1. Study design

This study used a cross-sectional research design to conduct a deep analysis of the survey data screened from our HIS (Hospital Information System) databases. We investigated the effects of preoperative frailty on POP among elderly patients undergoing surgical treatment for lower-extremity fractures.

### 2.2. Setting and population

This study enrolled patients From January 1, 2021 to June 30, 2022, at a tertiary hospital in China. A total of 568 elderly patients with lower-extremity fractures were admitted to the hospital during the research period. The inclusion criteria were age 60 years or older, main lower-extremity fracture, and surgical treatment. Exclusion criteria were age <60 years, open fracture, primary or secondary tumor-related fractures, phalangeal bone fracture, metatarsal bone fracture and tarsal bone fracture, with incorrect or incomplete information in the electronic medical record, and seriously ill patients transferred from another hospital after treatment. Our institution is a large, general, Grade III hospital. This study was approved by the Bioethics Committee of the People Hospital of Deyang City (No. LWH-OP006-A04-V2.0). This study did not receive any funding support, but informed consent was obtained from all the participants.

### 2.3. Instruments

#### 2.3.1. Clinical frailty scale.

The CFS is a tool widely used worldwide for the assessment of frailty in elderly patients. Previous studies have demonstrated the effectiveness of the CFS in predicting adverse outcomes.^[11]^ The CFS score ranges from 1 (very ft) to 9 (terminally ill). Each level is defined as follows: Level 1: very fit; Level 2: well; Level 3: managing well; Level 4: vulnerable; Level 5: mildly frail; Level 6: moderately frail; Level 7: severely frail; Level 8: very severely frail; and Level 9: terminally ill. In our study, CFS scores were stratified into 4 groups based on previously established cutoffs^[12]^: CFS 1 to 3 was “not frail,” CFS 4 was “vulnerable,” CFS 5 was “mildly frail,” and CFS 6 to 9 was “severely frail.”

#### 2.3.2. Demographic variables.

Age, gender, smoking history, and body mass index (BMI) were included after a literature review of relevant risk factors for POP. Age was classified into 2 categories: ˂75 years and ≥75 years. Smoking history was classified into 2 categories: current smoking or not. BMI was classified into 2 categories: ≥24 kg/m^2^ and ˂24 kg/m^2^.

#### 2.3.3. Health-related variables.

Hypoproteinemia, anemia, fracture site, hypertension, diabetes and chronic obstructive pulmonary disease (COPD) were included. We considered serum albumin levels <35 g/L as hypoproteinemia. Anemia was defined as a hemoglobin level <120g/L in men and 110 g/L in women. The fracture sites included hip fracture and non-hip fractures. Non-hip fracture refers to the main lower limb fractures except hip fracture, including femoral shaft fracture, distal femur fracture, patellar fracture, tibial plateau fracture, tibiofibular shaft fracture, ankle, and calcaneal fractures. Hypertension, diabetes and COPD was classified into 2 categories: Yes or No.

#### 2.3.4. Intervention-related variables.

Our department has set up a frailty management team, including 2 medical groups (groups A and B; each group was composed of 1 expert, 1 attending physician and 1–2 residents), 1 nursing team (composed of 1 deputy head nurse, 1 specialist nurse, and several nurse practitioners), and 1 scientific secretary. Patients were randomly assigned to either group A or Group B. Time from injury to surgery was classified into 2 categories: ˂72 hour or ˃72 hour. Type of anesthesia included general and introspinal anesthesia.

#### 2.3.5. Outcome variable.

Outcome variable was POP. As described in previous reports, POP was defined based on new or progressive radiographic infiltrate, bacterial sputum cultures, and 2 or more of the following: temperature > 38°C, antibiotic treatment, leukocytosis (white blood cell > 12 × 10^9^/L), or leukopenia (white blood cell count < 4 × 10^9^/L), or purulent secretions.^[13]^ The enrolled patients were categorized into 2 groups based on the presence of pneumonia at duration of hospital stay: with POP and without POP.

### 2.4. Sample size and data collection

According to previous reports, a rough estimation method of sample size was used: 10 times the number of study variables was used to calculate the sample size. There were 14 variables in the study, hence, the sample size of this study was 140 patients. Considering a loss rate of 20%, the minimum sample size of the study was 168 patients. We used Microsoft Excel 2010 software to collect data. Frailty was assessed immediately after admission. Other variables are retrieved from HIS system database.

### 2.5. Statistical analysis

Statistical analyses were conducted using SPSS 22.0 (SPSS, Inc., Chicago, IL) and GraphPad Prism 8.0 (GraphPad Prism Software Inc., San Diego, CA). There were no missing exposure/outcome data or losses to follow-up. Categorical variables were expressed as numbers (n) with percentages, while continuous variables were expressed as means with standard deviations. The chi-square test and Student *t* test were used for data analyses, as appropriate. Binary univariate and multivariate logistic regression analyses were used to determine the risk factors for POP using the “Enter” method. A multicollinearity analysis was performed to identify potential risk factors in the logistic regression model. Statistical significance was defined as a 2-sided *P* value < .05.

## 3. Results

The present study enrolled 568 patients according to the inclusion and exclusion criteria, and the patient characteristics stratified by the CFS score are shown in Table 1. All patients underwent frailty assessment within 24 hours after admission, and data on POP were not missing. The average age was 79.6 years old, and among all patients, 53.9% (306/568) were female, and 64.4% (366/568) were diagnosed with hip fracture. While 22.4% (127/568) of participants were not frail, 19.7% (112/568) were vulnerable, 21.3% (121/568) were mildly frail, and 36.6% (208/568) were severely frail.

**Table 1:** Characteristics of patients stratified by CFS score.

Variable	Not frail	Vulnerable	Mildly frail	Severely frail
Age, yr, mean (SD)	75.2 (7.0)	78.6 (8.3)	80.5 (7.9)	82.3 (8.4)
Gender				
Male, n (%)	62 (48.8)	53 (47.3)	55 (45.5)	92 (44.2)
Female, n (%)	65 (51.2)	59 (52.7)	66 (54.5)	116 (55.8)
Current smoking, n (%)	23 (18.1)	15 (13.4)	18 (14.9)	29 (13.9)
BMI, mean (SD)	21.6 (1.9)	22.7 (2.3)	22.9 (2.1)	21.5 (2.0)
Anemia, n (%)	86 (67.7)	83 (74.1)	91 (75.2)	174 (83.7)
Hypoproteinemia, n (%)	33 (26.0)	28 (25.0)	39 (32.2)	79 (38.0)
Group				
A, n (%)	67 (52.8)	53 (47.3)	66 (54.5)	105 (50.5)
B, n (%)	60 (47.2)	59 (52.7)	55 (45.5)	103 (49.5)
Time from injury to surgery				
<72 h, n (%)	23 (18.1)	15 (13.4)	11 (9.1)	9 (4.3)
>72 h, n (%)	104 (81.9)	97 (86.6)	110 (90.9)	199 (95.7)
Type of anesthesia				
General, n (%)	101 (79.5)	73 (65.2)	69 (57.0)	110 (52.9)
Introspinal, n (%)	26 (20.5)	39 (34.8)	52 (43.0)	98 (47.1)
Type of fracture				
Hip fracture, n (%)	54 (42.5)	93 (83.0)	67 (55.4)	152 (73.1)
Non-hip fracture, n (%)	73 (57.5)	19 (17.0)	54 (44.6)	56 (26.9)
Hypertension, n (%)	22 (17.3)	19 (17.0)	22 (18.2)	46 (22.1)
Diabetes, n (%)	22 (17.3)	23 (20.5)	30 (24.8)	40 (19.2)
COPD, n (%)	17 (13.4)	17 (15.2)	21 (17.4)	43 (20.7)

BMI = body mass index, CFS = clinical frailty scale, COPD = chronic obstructive pulmonary disease, SD = standard deviations.

As shown in Table 2, 65 patients developed pneumonia during postoperative hospitalization, accounting for 11.4% of all the elderly. There were no statistically significant differences in age, smoking, BMI, anemia, preoperative waiting time, fracture site, hypertension, or diabetes between the 2 groups (with and without POP). Male patients (*P* = .034), hypoproteinemia (*P* < .001), general anesthesia (*P* < .001), history of COPD (*P* < .001), and preoperative frailty (*P* = .002) were more likely to develop pneumonia after surgery. Notably, the proportion of POP was 12.8% in patients with hip fracture and 8.9% in patients with other main types of lower-extremity fractures. However, the difference between hip and non-hip fractures was not statistically significant (*P* > .05).

**Table 2:** Preoperative variables associated with postoperative pneumonia in elderly patients following surgical treatment of lower extremity fracture.

	Postoperative pneumonia (n = 65)	Without postoperative pneumonia (n = 503)	*P* value
Age			.407
≥75 yr, n (%)	43 (66.2)	306 (60.8)	
<75 yr, n (%)	22 (33.8)	197 (39.2)	
Gender			.034
Male, n (%)	38 (52.31)	224 (45.33)	
Female, n (%)	27 (47.69)	279 (54.67)	
Current smoking, n (%)	12 (18.46)	73 (14.51)	.401
BMI (kg/m^2^)			.956
≥24,n (%)	40 (61.54)	309 (61.43)	
<24, n (%)	25 (38.46)	196 (38.57)	
Anemia, n (%)	51 (78.46)	383 (76.14)	.679
Hypoproteinemia, n (%)	35 (53.85)	144 (28.63)	<.001
Group			.854
A, n (%)	34 (52.31)	257 (51.09)	
B, n (%)	31 (47.69)	246 (48.91)	
Time from injury to surgery			.476
<72 h, n (%)	5 (7.69)	53 (10.54)	
>72 h, n (%)	60 (92.31)	450 (89.46)	
Type of anesthesia			<.001
General, n (%)	56 (89.23)	297 (59.05)	
Introspinal, n (%)	9 (10.77)	206 (40.95)	
Type of fracture			.159
Hip fracture, n (%)	47 (72.31)	319 (63.42)	
Non-hip fracture, n (%)	18 (27.69)	184 (36.58)	
Hypertension, n (%)	13 (20.0)	96 (19.09)	.863
Diabetes, n (%)	16 (24.62)	99 (19.68)	.352
COPD, n (%)	39 (60.0)	59 (11.73)	<.001
CFS score			.002
Not frail (CFS 1–3), n (%)	5 (7.69)	122 (24.25)	
Vulnerable (CFS 4), n (%)	10 (15.38)	102 (20.28)	
Mildly frail (CFS 5), n (%)	14 (21.54)	107 (21.27)	
Frail (CFS 6–9), n (%)	36 (55.39)	172 (34.2)	

BMI = body mass index, CFS = clinical frailty scale, COPD = chronic obstructive pulmonary disease.

**Figure 1.**
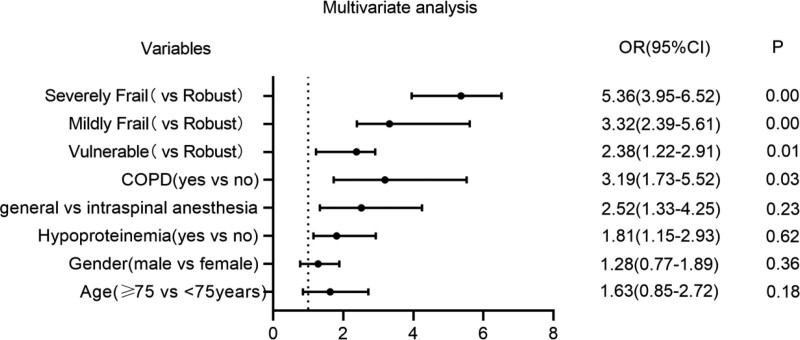
Forest plot of the multivariate logistic regression analysis for postoperative pneumonia.

Figure 1 shows that the adjusted multivariate regression analysis illustrated that the risk of POP was 1.63 (95% CI: 0.85–2.72, *P* = .18) times higher in patients aged 75 years and older and 1.28 (95% CI: 0.77–1.89, *P* = .36) times higher in males. The risk of POP in patients with hypoproteinemia increased by 1.81 (95% CI: 1.15–2.93, *P* = .62), and by 2.52 (95% CI: 1.33–4.25, *P* = .23) in patients who received general anesthesia. However, these differences were not statistically significant. Patients with COPD were 3.19 (95% CI: 1.73–5.52, *P* = .031) times more likely to develop POP than those with no history of COPD. Moreover, compared to patients without frailty, the risk of POP in vulnerable, mildly frail, and severely frail patients increased by 2.38 times [*P* = .01, 95% CI (1.22–2.91)], 3.32 [*P* = .00, 95% CI (2.39–5.61)], and 5.36 [*P* = .00, 95% CI (3.95–6.52)], respectively.

**Figure 2.**
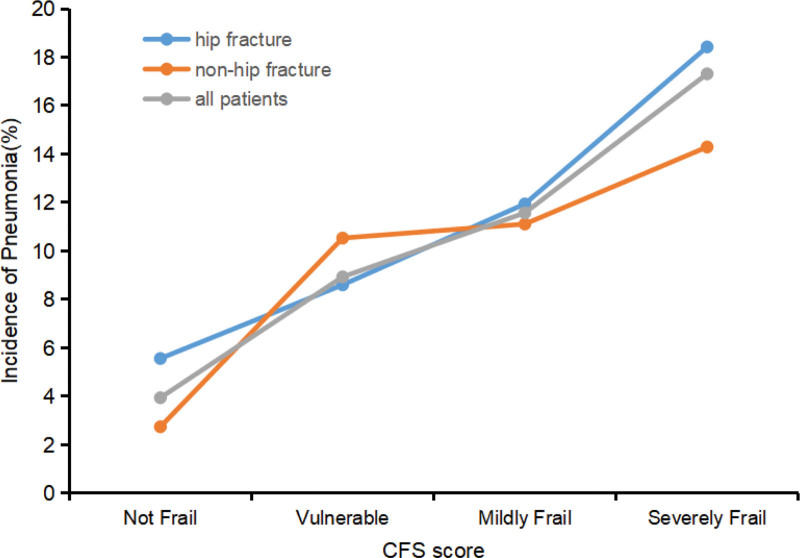
Line chart about incidence of postoperative pneumonia in elderly patients with different frail status.

Intergroup analyses were performed to assess the association between frailty and risk of POP in patients with hip and non-hip fractures. As shown in Figure 2, as frailty increased (not CFS score, but a frailty classification by CFS), the risk of POP increased almost linearly for both hip and non-hip fractures.

## 4. Discussion

### 4.1. Preoperative frailty increases the risk of POP in elderly patients with lower-extremity fractures

This study examined the cross-sectional association between frailty and risk of POP among elderly patients with lower-extremity fractures. The results showed that the CFS score was significantly and positively associated with the risk of POP after adjusting for potential confounders in elderly patients with lower-extremity fractures.

Our findings are consistent with those of previous studies. A retrospective cohort study^[9]^ (n = 965) demonstrated that preoperative frailty is independently associated with POP, and patients with frailty were more likely to develop in-hospital pneumonia than non-frail patients (20.6% vs 11.1%, χ^2^ = 9.24, *P* = .002), The multivariable analysis in the study showed that patients with frailty status statistically increased the risk of in-hospital pneumonia (adjusted odds ratio: 2.08; 95% CI: 1.28–3.39, *P* = .003). Another study reported an independent correlation between the modified Frailty Index-5 and POP.^[14]^

POP is a costly complication. However, there is little literature on risk factors, especially modifiable ones that drive complications. Although some studies have investigated the risk factors for POP in elderly patients with hip fractures,^[4,5,15]^ in-depth studies on the relationship between preoperative frailty and POP are still lacking. Moreover, whether frailty plays the same role in POP in patients without hip fractures (other major lower limb fractures) is unknown. The present study found that preoperative frailty also increased the risk of POP in patients with other major lower limb fractures not only in older patients with hip fractures. Our findings extend the scope of previous studies and are effective for all elderly patients with lower-extremity fractures undergoing surgical treatment. To some extent, this result is consistent with those of previous studies.^[16–18]^

The exact mechanisms and root causes from frailty to POP remain unclear. However, a few studies have suggested some plausible answers. First, frail patients with reduced motor function and sputum drainage disorders are prone to developing pneumonia. At the same time, studies have shown that after fractures, the internal environment of patients changes under stress, and some biochemical factors cause lung injury,^[19]^ which also increases the risk of pneumonia. In addition, excessive control of preoperative blood sugar levels in patients with diabetes not only causes malnutrition but also aggravates frailty,^[20]^ eventually increasing the risk of pneumonia. Frail patients are often malnourished and often have a poor diet, which presents a vicious cycle and leads to hypoproteinemia, and finally, low serum albumin levels increase the risk of pneumonia.^[21]^ In conclusion, multiple factors play a role in the high risk of POP in elderly patients with lower-extremity fractures.

### 4.2. CFS is effective and feasible for the preoperative assessment of frailty in elderly patients with lower limb fractures

There are many assessment scales for screening frailty in clinics. The present study is the first to use the CFS to assess frailty in elderly patients with lower-extremity fractures. Our results suggest that CFS can be used in traumatic orthopedics to screen elderly patients with lower limb fractures. Currently, the most commonly used scales for the assessment of frailty include the FI, frailty phenotype, and CFS. Given the theoretical background of a clear relationship between postoperative complications and preoperative frailty in elderly patients with lower limb-fractures, it is particularly critical to conduct a rapid and effective assessment of frailty. A previous study^[9]^ used the FI (frailty index) to study postoperative complications in elderly patients with hip fractures. FI is based on the principle of defect accumulation, and many items cannot be obtained when patients are admitted to the hospital; therefore, it cannot be used for prospective assessment. The frailty phenotype requires on-site patient evaluation, which is not possible in patients with lower-limb fractures. The use of CFS is simple and can be fully mastered and used by non-geriatric medical staff after simple training. It has been confirmed that CFS can be used in the assessment of frailty of the elderly in China,^[22]^ and it has also been reported in the field of orthopedics.^[23]^ The findings of our study were consistent with those of previous studies. It is of great significance to use CFS for the preoperative frailty assessment of elderly patients with lower-extremity fractures, which is critical for the development of clinical work.

Frailty is an overall manifestation of the decline of all system functions and the increase in vulnerability to urgent events with the increasing age; however, there is evidence that frailty is reversible and vulnerability is dynamic, which means that a person can fluctuate with the severity of vulnerability.^[24]^ Therefore, strategies to prevent and slow the progression of frailty are paramount.^[25]^

Previous studies have illustrated some interventions to prevent POP in elderly patients with fractures, such as wearing masks during the perioperative period,^[26]^ preoperative balloon blowing to exercise lung function, enhanced recovery after surgery in orthopedics, and establishing professional teams for hip treatment.^[27]^ Based on the association elaborated in this study, our future studies will focus on interventions targeting frailty which may provide a new direction for reducing POP in elderly patients with lower-extremity fractures.

## 5. Limitation

Our study had some limitations. First, the characteristics of the observational method determine the low level of evidence. Second, the present study is a single-center study, so the bias caused by medical technology exists. Moreover, the number of subjects in this study was small; thus, the real-world results are unknown.

## 6. Conclusion

Preoperative frailty increases the risk of POP in elderly patients after surgical treatment of main lower-extremity fractures. The severer the preoperative frailty is, the higher the risk of preoperative pneumonia is in elderly patients with lower-extremity fractures. CFS is simple and feasible for the assessment of frailty in elderly patients with lower-extremity fractures. Preoperative frailty assessment and appropriate management strategies should be considered in the perioperative management of elderly patients with lower-extremity fractures.

## Acknowledgments

We thank Wang Zhicong for helping with data collection, Peng Yumei, Tang Limei, Luo Meng, and Xiang Jiyao for helping with the assessment work, and Chen Xi for quality assurance of the research. Although all the members above did not participate in the writing of the article, they did a lot of help.

## Author contributions

**Conceptualization:** Yili Ou, Hong Wang.

**Data curation:** Wei Jiang.

**Formal analysis:** Wei Jiang.

**Methodology:** Ling Yang.

**Resources:** Hong Wang.

**Software:** Yili Ou.

**Validation:** Hong Wang.

**Writing – original draft:** Yili Ou.

**Writing – review & editing:** Yili Ou.
